# Supplementary material for: An explicit solution for calculating optimum spawning stock size from Ricker’s stock recruitment model
Source: PeerJ. 2016 Jan 25;4:e1623. doi: 10.7717/peerj.1623 (PMC4800783; doi:10.7717/peerj.1623)
Supplement: Figure S1 — See Table 1 in main text for an example of the function call. [file peerj-04-1623-s001.pdf]

1) Download **LambertWfunc.xlam** from https://github.com/mdscheuerell/Lambert-W-in-Excel or https://faculty.washington.edu/scheuerl/LambertWfunc.xlam and save it anywhere on your hard drive.

2) Start Excel.

3) Depending on your version of Excel, follow either (a) or (b) below.

   a) Microsoft Excel for Mac 2011:

      i) Click **Tools**, and then select **Add-ins...**

      ii) From the dialog box, click **Select…**

      iii) Browse to wherever you saved the file in Step (1) and select **LambertWfunc.xlam**.

      iv) Click **Open**, which returns you to the **Add-Ins** dialogue box.

      v) Verify the box is checked next to **LambertWfunc.xlam**.

      vi) Click **OK**.

   b) Microsoft Excel for Windows (versions 2007, 2010, 2013)

      i) Click the **Office Button** (v2007) or the **File** tab (v2010/2013).

      ii) Click on **Options** near the bottom of the list.

      iii) From the pop-up window, choose the **Add-Ins** category.

      iv) In the **Manage** box at the bottom, click **Excel Add-ins**, and then click **Go**.

      v) In the **Add-Ins** dialog box that appears, click **Browse**.

      vi) Browse to wherever you saved the file in Step (1) and select **LambertWfunc.xlam**.
